# Use of RESERVE-Antibiotics in Newborns: Clinical Experience of Two NICUs in the Metropolitan Area of Palermo

**DOI:** 10.3390/antibiotics15020231

**Published:** 2026-02-21

**Authors:** Veronica Notarbartolo, Deborah Bacile, Bintu Ayla Badiane, Agnese Lo Leggio, Vita Maria Angileri, Vincenzo Duca, Mario Giuffré

**Affiliations:** 1 Neonatology and Neonatal Intensive Care Unit, University Hospital “Paolo Giaccone”, 90127 Palermo, Italy; mario.giuffre@unipa.it; 2 Department of Health Promotion, Mother and Child Care, Internal Medicine and Medical Specialties, University of Palermo, 90127 Palermo, Italy; deborah.bacile@community.unipa.it (D.B.); bintuayla.badiane@community.unipa.it (B.A.B.); agnese.loleggio@community.unipa.it (A.L.L.); 3 Neonatal Intensive Care Unit with Neonatology, “G.F. Ingrassia” Hospital Unit, 90131 Palermo, Italy; vita.angileri@asppalermo.org (V.M.A.); vincenzo.duca@asppalermo.org (V.D.)

**Keywords:** ceftazidime-avibactam, newborns, preterms, NICUs, antibiotics, meropenem-vaborbactam, ceftolozane-tazobactam, sepsis, RESERVE

## Abstract

**Background**: The increasingly indiscriminate use of antibiotic therapy in the neonatal period has led to the emergence of multidrug-resistant organisms (MDROs), which are responsible for sepsis that is increasingly difficult to treat and associated with high morbidity and mortality. Increasingly frequently, in neonatal intensive care units (NICUs), it is necessary to use last-generation antibiotics belonging to the RESERVE group according to the current classification of the World Health Organization (WHO). **Methods**: Among these drugs, ceftazidime-avibactam, ceftolozane-tazobactam and meropenem-vaborbactam are increasingly used in infections caused by Enterobacterales (i.e., *E. cloacae complex*, *Klebsiella* spp.), which are often responsible for late-onset sepsis (LOS) in newborns, especially in preterms. **Results**: Here, we present the experience of four newborn patients in the city of Palermo, treated over a period of 3 years. **Conclusions**: The comparison between different diagnostic–therapeutic management approaches and a review of the most recent literature can contribute to identifying more standardized pharmacological schemes, especially in the neonatal period, where scientific evidence about this topic is still very limited.

## 1. Introduction

Neonatal sepsis persists as a primary contributor to morbidity and mortality in newborns, particularly among preterms. Despite the development of various approaches for prompt diagnosis and management of the condition, the occurrence rate of Late-Onset Sepsis (LOS) has failed to decrease, leading to a rising prevalence of antibiotic resistance [1]. Nosocomial infections sustained by multidrug-resistant microorganisms (MDROs), such as Enterobacterales, represent a global public health threat, a major therapeutic challenge and a significant cause of morbidity and mortality in the neonatal setting, causing over 35,000 deaths annually in Europe, particularly among critically ill infants with surgical comorbidities [2]. These microorganisms are known for their remarkable ability to rapidly acquire antimicrobial resistance, limiting available therapeutic options and leading healthcare workers to use, often off-label, new generation drugs belonging to the RESERVE antibiotic group [3]. Among these, there are cephalosporins, such as ceftazidime/avibactam (CAZ-AVI), which amalgamates a third-generation cephalosporin with a non-β-lactam β-lactamase inhibitor, and ceftolozane-tazobactam, which is a new-generation cephalosporin associated with a β-lactam β-lactamase inhibitor [4]. Also, meropenem-vaborbactam belongs to this antibiotic category; it includes a carbapenem and a non-β-lactam β-lactamase inhibitor. These drugs may be considered in selected cases of severe infections, when commonly used antibiotics for the neonatal age group are not effective [5]. This article describes four cases of premature neonates with LOS caused by extensively drug-resistant Enterobacterales, where the off-label use of the latest-generation antibiotics represents the last resort in complex and complicated clinical contexts.

## 2. Results

### 2.1. Case 1

We describe the case of a premature male neonate, the second twin from a monochorionic diamniotic pregnancy, born in a first-level hospital setting at 35^+3^ weeks via emergency cesarean section for fetal heart rate decelerations. At birth, abundant salivary secretions and inability to advance the orogastric tube beyond the pharynx raised suspicion of esophageal atresia, subsequently confirmed by radiological assessment. Chest X-ray revealed arrest of the orogastric tube at the level of T1 with a loop formation; the presence of distal bowel gas supported the suspicion of a distal tracheoesophageal fistula. The infant was then transferred to the University Hospital “P. Giaccone” for specialized management. Upon admission, in view of the suspected diagnosis, a tube was positioned for continuous aspiration. Ultrasound screenings were performed: echocardiography revealed a patent *foramen ovale* and a perimembranous ventricular septal defect (0.5 × 0.4 cm) with left-to-right shunt; cranial and abdominal ultrasounds were otherwise unremarkable. On day of life (DOL) 6, the infant underwent early surgical correction with ligation of the distal tracheoesophageal fistula, gastrostomy creation, and thoracic drainage. Due to the wide gap between the two esophageal ends, primary anastomosis was not feasible. The initial postoperative course was satisfactory. On postoperative day 10, a septic picture emerged, characterized by high fever, severe thrombocytopenia, and elevated inflammatory markers (C-Reactive Protein, CRP > 30 mg/L). Blood cultures grew methicillin-resistant *Staphylococcus aureus* (MRSA), and a targeted therapy with vancomycin was initiated, with subsequent clinical improvement. After 48 h, a new inflammatory flare occurred, with rising CRP (113 mg/L) and worsening thrombocytopenia (19,000/mm^3^). Blood culture and cultures from the removed thoracic drain yielded *Enterobacter hormaechei*, prompting the initiation of amikacin in addition to vancomycin, resulting in temporary stabilization. Following a new episode of clinical deterioration with high fever (up to 39.1 °C), petechiae, and increased inflammatory indices, repeat blood cultures were positive for *Enterobacter cloacae complex*, capable of inactivating third-generation cephalosporins (PCR CTX-M (Cefotaximase-Munich), positive). After a multidisciplinary meeting, on postoperative day 14, the procedure for off-label administration of CAZ-AVI was activated, using a dosage of 25 (20/5) mg/kg/dose every 8 h, infused over 2 h. A subsequent blood culture again yielded *E. hormaechei*. The antibiogram also showed susceptibility to amikacin; therefore, considering the severity of the clinical picture, polypharmacological therapy was started, and CAZ-AVI + aminoglycoside was administered *ex-adiuvantibus*. After 48 h, an initial reduction in inflammatory markers, hemodynamic stabilization, and subsequent blood culture clearance were observed. Dual antibiotic therapy was discontinued after 14 days in light of clinical, laboratory, and microbiological improvement. At approximately one month of life (46 DOL), the infant underwent esophageal end-to-end anastomosis, but the persistent distance between the two esophageal stumps and the difficulty in mobilizing the upper one from the thoracic inlet caused massive hemothorax, resulting in the death of the young patient a few hours after surgical intervention.

### 2.2. Case 2

We report the case of a premature female neonate born at 33^+4^ weeks via precipitous vaginal delivery, with prenatal findings suggestive of congenital intestinal malformation (polyhydramnios and gastric/jejuno-ileal distension). At birth, the infant developed severe respiratory distress requiring intubation and surfactant administration. Initial abdominal X-ray showed marked gastric and proximal small bowel distension with an absence of distal gas, consistent with intestinal atresia. Clinical assessment revealed abdominal distension, bilious nasogastric output, and multiple dysmorphic features. Pediatric surgical evaluation, combined with the imaging findings, confirmed the suspicion of jejunal and/or ileal atresia; therefore, the newborn was transferred to the University Hospital “P. Giaccone” for specialized management. On DOL 2, the infant underwent surgery with two end-to-end anastomoses and resection of approximately 37.5 cm of intestine. Screening cranial and abdominal ultrasounds were normal. Echocardiography revealed an *ostium secundum* atrial septal defect (5 mm) with mild-to-moderate mitral and tricuspid regurgitation. Histopathology of the resected bowel showed complete luminal atresia with vascular fibrosis and preserved ganglion cells, excluding congenital aganglionosis. The initial postoperative course was stable, with early extubation and adequate vital parameters. Subsequent hyperbilirubinemia was treated with phototherapy, and generalized edema required albumin infusion and diuretic therapy. On DOL 16, persistent bilious gastric drainage and absent meconium passage prompted further contrast evaluation: upper GI study demonstrated duodenal dysmotility with persistent ectasia, while contrast enema showed obstruction at the sigmoid colon suggestive of colonic atresia. A second laparotomy confirmed a short (1.5 cm) segment of colonic atresia, which was resected with primary anastomosis. Meconium passage occurred within 48 h postoperatively, and gradual enteral feeding was attempted but repeatedly interrupted due to bilious vomiting. On DOL 20, the infant developed severe sepsis with acute respiratory distress, leading to initiation of empirical vancomycin and amikacin. Persistent irritability, hypertonia, and opisthotonic posturing raised suspicion of central nervous system (CNS) involvement; cerebrospinal fluid examination was normal. Abdominal ultrasound revealed hepatosplenomegaly, ascites, and increased renal echogenicity. Chest X-ray showed diffuse interstitial thickening progressing to left pleural effusion with atelectasis. Blood and central line cultures repeatedly yielded MDR *Klebsiella pneumoniae* (PCR KPC (*Klebsiella Pneumoniae* carbapenemase) and PCR CTX-M positive). Antibiotic therapy was sequentially modified: first to fosfomycin, able to cross the blood–brain barrier (BBB), then, after the exclusion of neurological involvement, meropenem-vaborbactam was administered at 40 (20/20) mg/kg/dose, every 8 h, over 3 h. After 48 h, following the onset of antimicrobial resistance according to the antibiograms, CAZ-AVI was administered at 25 (20/5) mg/kg/dose every 8 h over 2 h, with close renal monitoring, especially when amikacin was added. Despite advanced antimicrobial therapy, inotropic support, transfusions, and invasive mechanical ventilation, the infant developed progressive hepatic failure and multiorgan dysfunction, resulting in death at DOL 63.

### 2.3. Case 3

A 718 g, 25-week and 5-day male infant was born to a 29-year-old diabetic female with treated hypothyroidism by emergency cesarean delivery due to premature rupture of membranes and oligohydramnios caused by maternal infection at “G. F. Ingrassia” Hospital. At birth, according to the maternal anamnesis, empiric prophylactic antibiotic therapy with ampicillin and amikacin was started, then discontinued after a negative blood culture result (DOL 5). On DOL 6, he showed clinical instability characterized by apnea and low oxygen saturation. He presented anemia, hematemesis and coagulopathy; therefore, he received red blood cell transfusion, vitamin K i.m. and antithrombin III i.v. An echocardiogram was also done and showed hemodynamically significant patent ductus arteriosus (PDA), and a cycle of ibuprofen was started. The newborn also showed hyperglycemia that was treated with continuous insulin infusion. On DOL 10, his clinical condition worsened again. Blood culture and cell-blood count (CBC) were drawn, and empirical antibiotic therapy with teicoplanin and ceftazidime was started. Due to hemodynamic instability, vasoactive amines were administered. On DOL 12, due to respiratory distress and poor clinical condition, he was placed under invasive mechanical ventilation. Antibiotic therapy was switched again to piperacillin-tazobactam + amikacin. On DOL 13, blood culture showed the presence of *Klebsiella pneumoniae* MDR (PCR KPC-positive) and, according to the antibiogram, piperacillin-tazobactam was switched to ceftolozane-tazobactam at a dosage of 18 (12/6) mg/kg/dose every 8 h over 1 h. The echocardiogram was repeated and still showed hemodynamically significant PDA, so a cycle of paracetamol was started. On DOL 16, the newborn was extubated and placed under non-invasive ventilation. He also had cholestatic jaundice due to prolonged parenteral nutrition; therefore, treatment with ursodeoxycholic acid was started. On DOL 20, due to another deterioration of his condition, blood tests and culture were drawn and ceftolozane-tazobactam was replaced by ceftazidime-avibactam at a dosage of 50 (40/10) mg/kg/dose every 8 h over 2 h. The new blood culture was still positive for multidrug-resistant *K. Pneumoniae*. Blood tests showed a severe thrombocytopenia (20,000/mm^3^). During infectious episodes, due to severe anemia, thrombocytopenia and coagulopathy, several transfusions of red blood cells, platelets and plasma were administered. On DOL 23, the infant died due to intestinal perforation.

### 2.4. Case 4

A 1185 g, 30-week and 5-day male infant was born to an epileptic female with proteinuria and hypertension by emergency cesarean delivery for severe Intrauterine Growth Restriction (IUGR) and flowmetry alteration at “G. F. Ingrassia” Hospital. At birth, the infant presented with respiratory distress, so blood culture and a CBC were drawn, then empirical ampicillin and amikacin antibiotic therapy was started, which was interrupted after 7 days due to negative results from a blood culture. On DOL 13, he presented with a poor clinical condition. Laboratory results showed a picture of sepsis, with leukopenia and increased inflammation indices (CRP was 10.89 mg/dL and procalcitonin was 22.97 ng/L). Empirical antibiotic therapy with ceftazidime, teicoplanin and metronidazole was started. The blood culture result was positive for *Enterobacter cloacae* (PCR CTX-M positive). According to the antibiogram, antibiotic therapy was switched to meropenem, ceftazidime-avibactam (50 (40/10) mg/kg/dose, every 8 h, over 2 h) and gentamicin. Despite the negativization of blood culture at DOL 20, antibiotic therapy was continued, but was finally stopped on DOL 68 due to the fact that LOS was complicated by meningoencephalitis (cerebrospinal fluid culture positive for *E. cloacae*) and several brain abscesses that led to post-infectious hydrocephalus. Neurosurgeons performed several transfontanellar drains and administered intrathecal gentamicin, according to the antibiogram. Finally, ventricular reservoirs were placed. During his stay, the infant showed seizures that were treated with anticonvulsants, and he needed several red blood cell transfusions due to anemia. He was discharged at 4 months of life with indication for neurological and neurosurgical follow-up.

In Table 1, a schematic representation is presented of the isolated microorganisms and the therapeutic regimens used.

In Table 2, clinical courses of neonates are described [**1**, modified].

## 3. Discussion

LOS remains one of the leading causes of morbidity and mortality in NICUs, with MDR organisms playing an increasingly prominent role. Among MDROs, *Enterobacterales* (i.e., *K. pneumoniae*, *Enterobacter cloacae complex*) represent a major therapeutic challenge due to the limited efficacy of standard antibiotic regimens and poor clinical outcomes, particularly in preterm infants and those with surgical comorbidities [6,7]. The cases we presented highlight the complexity of managing neonates, particularly those who are preterm and/or have congenital malformations requiring surgical intervention, making them particularly vulnerable to severe nosocomial infections. The off-label use of Ceftazidime/Avibactam (CAZ-AVI) was necessary in all four patients as a potentially life-saving treatment, although adequate control of the infection was not always achieved. None of the four treated newborns experienced adverse events related to the use of this drug, underlining its good tolerance and safety profile. These cases underscore the growing urgency for new therapeutic options against multidrug-resistant pathogens in the neonatal setting and the need for further data on the use of CAZ-AVI in this age group. The available literature on CAZ-AVI use in neonates remains extremely limited, but it is expanding. Emerging studies and reports suggest a potential role for this molecule in infections caused by multidrug-resistant Gram-negative bacteria [1,8,9,10,11,12]. Since 2019, the European Medicines Agency (EMA) and the Food and Drug Administration (FDA) have approved the use of CAZ-AVI in infants aged at least 3 months for the treatment of complicated intra-abdominal infections, complicated urinary tract infections, and hospital-acquired or ventilator-associated pneumonia [11,12]. Some authors have reported the use of CAZ-AVI in meningitis, confirming the drug’s potential efficacy in treating severe neonatal infections caused by MDR pathogens, including the possibility of meningeal involvement (estimated around 38%) [13]. However, the prognosis remains strongly influenced by the patient’s baseline clinical condition and the timeliness of therapeutic intervention [14]. According to Asfour et al. [1], the pathogens most frequently implicated in LOS are coagulase-negative *Staphylococcus*, *Escherichia coli*, *Klebsiella* spp., *Enterobacter cloacae complex*, and *Pseudomonas aeruginosa*, each with a prevalence of approximately 18.5%, while *Serratia marcescens* and *Acinetobacter* spp. are less common. The annual incidence of MDR-Enterobacterales infections is estimated at around 0.27–0.3%, with a globally increasing trend, as confirmed by recent epidemiological analyses and systematic reviews [15]. Among neonatal cases of infection caused by multidrug-resistant microorganisms, four required treatments with RESERVE antibiotics, two of them were caused by *K. pneumoniae,* and the other two by *Enterobacter cloacae complex*. Infections caused by MDR *K. pneumoniae* are notable for their severity and therapeutic challenges, particularly in preterm neonates or surgical patients, as in the clinical cases we described. Several case reports support these findings and have paved the way for off-label use of CAZ-AVI in complex clinical settings, although the literature remains limited to a few isolated cases. Regarding safety, rare adverse events have been reported, including worsening renal function, although a clear causal relationship with the drug has not been established [16,17,18]. A recent narrative review [19] described the role of CAZ-AVI (alongside Ceftolozane/Tazobactam and MER-VAB) in the treatment of neonatal sepsis caused by multidrug-resistant Gram-negative bacteria, reporting a favorable efficacy and tolerability profile in NICU populations. Additional cases and small series, including a record of Italian experiences in preterm neonates [9], have reported positive outcomes without significant toxicity. On a broader scale, a systematic review focusing on next-generation antimicrobials for neonatal sepsis identified CAZ-AVI among all RESERVE antibiotics as the most frequently reported agent in clinical neonatal cases [5], with approximately 90% clinical and microbiological success. Dermitzaki et al. [19] reported that approximately 31% of fatal neonatal sepsis cases are caused by MDR pathogens, particularly Enterobacterales and *Pseudomonadales*. CAZ-AVI has been shown to be active against most members of Enterobacterales, including extended-spectrum β-lactamase (ESBL) producers, AmpC producers, and class D carbapenemase-producing strains. The most recent evidence in the neonatological field comes from the phase 2a study by Bradley et al. [11], which evaluated the safety of CAZ-AVI in neonates from birth to three months of age, including preterm infants ≥ 31 weeks of gestational age. Preliminary results indicate a favorable tolerability profile, good pharmacokinetics, and no serious drug-related adverse events [3]. Recommended dosing is 25 (20/5) mg/kg per dose, every 8 h, over 2 h, for neonates ≤ 28 days old or preterm infants < 44 weeks postmenstrual age (PMA), and 37.5 (28/9.5) mg/kg per dose, every 8 h, over 2 h, for more mature infants. However, limitations and uncertainties remain [17]. In the first two clinical cases presented, this dosage was followed; conversely, in the other two, the severe clinical conditions associated (case 4) with neurological involvement led practitioners to opt for the approved pediatric dosage >3 months [20]. In vitro efficacy may vary depending on the type of carbapenemase, with reduced activity against metallo-β-lactamases [21]. The optimal dosing in neonates, especially preterm infants, has yet to be clearly defined [22]. Additional data come from larger populations [23]: a retrospective study of patients aged 0–18 years with infections caused by resistant organisms showed an 84% clinical response and a 30-day mortality of 20%, further supporting the potential role of CAZ-AVI in critically ill pediatric patients. Finally, a recent systematic review [5] on new antimicrobial therapies for neonatal sepsis reiterated the need for dedicated protocols and specific pharmacokinetic studies to define dosing and indications in the neonatal population. Combination with an aminoglycoside (gentamicin or amikacin), as in cases 2, 3 and 4, to enhance antibacterial activity against multidrug-resistant strains has been discussed in the literature, highlighting the importance of monitoring renal function during such combination therapy due to the risk of aminoglycoside-induced nephrotoxicity [24]. Nevertheless, polypharmacy would not be associated with a better outcome than monotherapy with CAZ-AVI alone.

In contrast, clinical experience with meropenem-vaborbactam (MER-VAB) in the neonatal setting is almost entirely lacking [22,25]. As highlighted by Poggi et al. [5], there are no reports of use in this population, although the drug demonstrates high in vitro efficacy against class A carbapenemase-producing strains (KPC) and shows potential therapeutic interest for severe infections caused by Carbapenem-resistant Enterobacterales. In the case 2 reported here, MER-VAB was attempted sequentially before the introduction of CAZ-AVI, but without evidence of sustained clinical benefit and only for a short period, due to the onset of drug resistance (48 h). The dosage used was chosen in accordance with the few phase 1 studies in the pediatric population that are currently available, in line with known principles of pharmacodynamics and pharmacokinetics (>1 month of life) [26]. Regarding the use of Ceftolozane–Tazobactam, it emerges as a potential additional therapeutic option in neonates, as it is approved for the treatment of complicated urinary tract infections from birth and supported by phase 1 and 2 clinical studies demonstrating a favorable safety profile and efficacy comparable to that of meropenem, particularly in the presence of multidrug-resistant Gram-negative pathogens [27]. In case 3, the drug was used temporarily at the lowest permitted pediatric dosage, as the newborn being treated was preterm < 32 weeks [28]. Currently, its use is not widely supported in the neonatal period.

In our cases, off-label use of these drugs was justified by three main factors: lack of effective and safe alternative therapies, clinical deterioration with hemodynamic instability and cytopenia, and documented susceptibility of the pathogen in the antibiogram.

In summary, our cases fit within an evolving clinical and scientific context: among RESERVE-antibiotics, CAZ-AVI seems to represent a promising therapeutic option for neonates with severe infections caused by multidrug-resistant Gram-negative bacteria. However, it is essential to expand the evidence base through prospective studies on pharmacokinetics, safety, and efficacy, in order to develop appropriate guidelines for this highly vulnerable population.

The main limitations of the study are: the small number of cases, the different hospital settings with consequential heterogeneity of the presented cases and treatment regimens, the presence of major confounding factors (i.e., extremely prematurity, surgical procedures, genetic anomalies), the fact that CAZ-AVI activity varies according to resistance mechanisms, different in different care settings, the inability to perform a systematic adverse event assessment, particularly given the frequent concomitant use of potentially nephrotoxic agents (i.e., aminoglycosides).

## 4. Materials and Methods

We present a collection of four clinical cases from two NICUs in the city of Palermo, the University Hospital “P. Giaccone” and the “G. F. Ingrassia” Hospital, collected over a period of 3 years (2023–2025). We also revised the most recent literature about this topic (2019–2025). English clinical trials, original papers, reviews, case reports, and meta-analyses were included. The following keywords (alone or in combination) were used: antibiotics, ceftazidime-avibactam; newborns; preterm; NICUs; meropenem-vaborbactam; ceftolozane-tazobactam; sepsis; RESERVE. The electronic databases used were PubMed and Scopus. Informed consent was obtained from the parents of all subjects involved in the study. The ethics committee approved the off-label use of the drugs.

For rapid pathogen identification in blood, the BioFire^®^ FilmArray^®^ Blood Culture Identification (BCID, USA) Panel kit was used. Culture and plating were then performed using the BD BACTEC™ FX automated system (Becton Dickinson, USA). Antibiograms were interpreted according to the most recent EUCAST criteria [29]. The maximum incubation time to define a blood culture as negative was 120 h.

## 5. Conclusions

The cases described highlight the complexity of care for neonates with multidrug-resistant nosocomial infections, which require multidisciplinary management, sequential therapeutic approaches, and intensive post-operative monitoring. In the neonatal period, therapeutic options against carbapenemase-producing bacteria are extremely limited. In light of the increasing incidence of MDRO infections in the neonatal period, there is an urgent need for prospective multicenter studies and international registries to define the efficacy and safety profiles of these new antibacterial agents, as well as their impact on long-term outcomes. To date, a comparative analysis of the literature data highlights that Ceftazidime-Avibactam might represent one of the most promising options for the treatment of neonatal infections caused by multidrug-resistant Enterobacterales, with the largest number of studies and associated results; otherwise, Meropenem-Vaborbactam and Ceftolozane-Tazobactam require further studies to define their safety profile and efficacy in this population. In this direction, knowledge of any intrinsic resistance genes that can be limited by the combined use of drugs is certainly helpful.

## Figures and Tables

**Table 1:** Case-reports: demographic and epidemiological characteristics.

	Case 1	Case 2	Case 3	Case 4
**Demographic characteristics**	Male, 35^+3^, twin, affected by esophageal atresia (long-gap) + fistula	Female, 33^+4^, affected by intestinal atresia	Male, 25^+5^	Male, 30^+5^
**First episode of sepsis**	*Staphylococcus aureus* MRSA (DOL 13), in blood culture	MDR *Klebsiella pneumoniae*, in blood culture (DOL 20)	MDR *Klebsiella pneumoniae*, in blood culture (DOL 13)	*Enterobacter cloacae*, in blood culture (DOL 13)
**Antibiotic used**	Vancomycin	Vancomycin + Amikacin	Teicoplanin + Ceftazidime (for 48 h, suspended for limited clinical benefit); Piperacillin-Tazobactam + Amikacin (for 48 h); Ceftolozane- Tazobactam + Amikacin	Ceftazidime + Teicoplanin + Metronidazole (for 48 h, until the result of antibiogram). Meropenem + Ceftazidime-Avibactam + Gentamicin
**Dosage**	15 mg/kg/dose every 8 h	15 mg/kg/dose every 8 h; 15 mg/kg/die	8 mg/kg/die (after loading dose of 16 mg/kg/die) + 30 mg/kg/dose every 8 h; 100 mg/kg/dose, every 8 h + 15 mg/kg/die; 18 (12/6) mg/kg/dose, every 8 h, over 1 h + 15 mg/kg/die.	30 mg/kg/dose every 8 h + 8 mg/kg/die (after loading dose of 16 mg/kg/die) + 7.5 mg/kg/dose every 12 h (after loading dose of 15 mg/kg). 40 mg/kg/dose, every 8 h + 50 (40/10) mg/kg/dose, every 8 h, over 2 h + 5 mg/kg every 36 h, then every day
**Second episode of sepsis**	*Enterobacter cloacae complex* (i.e., *E. hormaechei*), DOL 18, in blood culture and thoracic drain culture	MDR *Klebsiella pneumoniae*, in blood culture and central catheter culture (DOL 30)	MDR *Klebsiella pneumoniae*, in blood culture (DOL 20)	*Enterobacter cloacae*, in cerebrospinal fluid (CSF) culture (DOL 24)
**Antibiotic used**	Ceftazidime-Avibactam (CAZ-AVI) + Amikacin	Fosfomycin (discontinued after exclusion of neurological involvement); Meropenem-Vaborbactam (for 48 h, due to emergence of antimicrobial resistance); Ceftazidime-Avibactam (CAZ-AVI); Amikacin	Ceftazidime-Avibactam (CAZ-AVI)	Ceftazidime-Avibactam (CAZ-AVI) + intrathecal administration of Gentamicin
**Dosage**	25 (20/5) mg/kg/dose, every 8 h, infused over 2 h; 15 mg/kg/die	50 mg/kg/dose, every 12 h; 40 (20/20) mg/kg/dose, every 8 h, over 3 h; 25 (20/5) mg/kg/dose, every 8 h, infused over 2 h; 15 mg/kg/die	50 (40/10) mg/kg/dose, every 8 h, over 2 h	50 (40/10) mg/kg/dose, every 8 h, over 2 h + 5 mg/kg/die
**Timing of CAZ-AVI**	14 days, discontinued after two negative blood cultures	27 days, until death (DOL 63)	3 days, until death (DOL 23)	50 days (DOL 68), after two negative CSF cultures and resolution of intrathecal abscesses

DOL: days of life. The dosage of commonly used drugs was chosen in relation to the treatment protocols provided by the app of the Italian Society of Neonatology (NEOFARM SIN ^(R)^).

**Table 2:** Case-reports and climical courses.

DOL	Culture	Organism	Antibiotics
Clinical Course of Neonate Described: Case 1			
0	Blood	No growth	Empirical Ampicillin + Gentamicin antibiotic therapy for 7 days
13	Blood	Methicillin-resistant *Staphylococcus aureus* (MRSA)	Start of Vancomycin therapy
13	Thoracic drain	*Enterobacter hormaechei*	Vancomycin + Amikacin
18	Blood	*Enterobacter cloacae complex* (ECC)	Stop Vancomycin, start CAZ-AVI + Amikacin therapy
19	CVC	*Enterobacter cloacae complex* (ECC)	The same as before
21	Blood	No growth	The same as before
24	Blood	*Enterobacter hormaechei*	The same as before
31	Blood and CVC	No growth	The same as before
32	Blood	No growth	Stop antibiotic therapy
Clinical Course of Neonate Described: Case 2			
0	Blood	No growth	Empirical Ampicillin + Gentamicin antibiotic therapy for 7 days
20	Blood	MDR *Klebsiella pneumoniae*	Start Vancomycin + Amikacin therapy
30	Blood and CVC	MDR *Klebsiella pneumoniae*	Stop Vancomycin, start Fosfomycin + Amikacin
32	CSF	No growth	Stop Fosfomycin and Amikacin, start Meropenem-Vaborbactam (MER-VAB)
35	Blood	MDR *Klebsiella pneumoniae*	Stop MER-VAB, start CAZ-AVI therapy
40	Blood	MDR *Klebsiella pneumoniae*	The same as before
44	Blood and thoracic aspiration	MDR *Klebsiella pneumoniae*	CAZ-AVI + Amikacin
50	Blood and CVC	MDR *Klebsiella pneumoniae*	The same as before
60	Blood	MDR *Klebsiella pneumoniae*	Stop with death
Clinical Course of Neonate Described: Case 3			
0	Blood	No growth	Empirical Ampicillin + Amikacin antibiotic therapy for 5 days
13	Blood	MDR *Klebsiella pneumoniae*	Start Teicoplanin + Ceftazidime therapy for 48 h, then stop and start Piperacillin-Tazobactam + Amikacin
15	Blood and CVC	MDR *Klebsiella pneumoniae*	Stop Piperacillin-Tazobactam and start Ceftolozane-Tazobactam + Amikacin
20	Blood	MDR *Klebsiella pneumoniae*	Stop Ceftolozane-Tazobactam + Amikacin and start CAZ-AVI
23	Blood	MDR *Klebsiella pneumoniae*	Stop with death
Clinical Course of Neonate Described: Case 4			
0	Blood	No growth	Empirical Ampicillin + Amikacin antibiotic therapy for 5 days
13	Blood	*Enterobacter cloacae*	Start Teicoplanin + Ceftazidime + Metronidazole therapy for 48 h, then stop and start Meropenem + CAZ-AVI + Gentamicin
20	Blood	No growth	Stop Meropenem
24	CSF	*Enterobacter cloacae*	CAZ-AVI + intrathecal Gentamicin
30	CSF	*Enterobacter cloacae*	The same as before
40	CSF	*Enterobacter cloacae*	Tha same as before
50	Blood/CSF	No growth	The same as before
68	CSF	No growth	Stop therapy

DOL: days of life; MRSA: methicillin-resistant *Staphylococcus epidermidis*; ECC: *Enterobacter cloacae complex*: CAZ-AVI: Ceftazidime/Avibactam; CVC: central venous catheter. DOL: days of life; CAZ-AVI: Ceftazidime/Avibactam; CVC: central venous catheter; CSF: cerebrospinal fluid; MDR: multi-drug resistant; MER-VAB: Meropenem-Vaborbactam. DOL: days of life; CAZ-AVI: Ceftazidime/Avibactam; CVC: central venous catheter; MDR: multi-drug resistant. DOL: days of life; CAZ-AVI: Ceftazidime/Avibactam; CSF: cerebrospinal fluid.

## Data Availability

The original contributions presented in this study are included in the article. Further inquiries can be directed to the corresponding author(s).
